# Oestrogen receptor expression distinguishes non-ossifying fibroma from other giant cell containing bone tumours

**DOI:** 10.1007/s00428-022-03341-4

**Published:** 2022-05-25

**Authors:** Arjen H. G. Cleven, Willem H. Schreuder, Eline Groen, Jan de Lange, Inge H. Briaire-de Bruijn, Judith V. M. G. Bovée

**Affiliations:** 1 Department of Pathology, Leiden University Medical Center, Albinusdreef 2, 2333 ZA Leiden, The Netherlands; 2 Department of Oral and Maxillofacial Surgery / Head and Neck Surgery, Amsterdam University Medical Center/Antoni Van Leeuwenhoek Hospital, Amsterdam, The Netherlands

**Keywords:** ERα expression, NOF, CGCG

## Abstract

Non-ossifying fibroma (NOF) and central giant cell granuloma (CGCG) are both benign tumours of bone with overlapping morphology and similar mutations in the RAS/MAPK pathway. However, NOF is located in the long bones with regression after puberty in contrast to CGCG which is located in the jaw bones and does not regress spontaneously. We hypothesised that endocrine regulation by oestrogen plays a role in the spontaneous regression in NOF. Therefore, we examined the expression of ERα in a series of NOF and CGCG. ERα expression (EP1) was determined using immunohistochemistry on 16 NOFs (whole slides), and 47 CGCGs (tissue microarrays (TMA’s *n* = 41 and whole slide *n* = 6)). As comparison, we included TMAs of other giant cell containing bone lesions: giant cell tumour of bone (*n* = 75), chondroblastoma (*n* = 12), chondromyxoid fibroma (*n* = 12), aneurysmal bone cyst (*n* = 6) and telangiectatic osteosarcoma (*n* = 6). All 16 NOF samples demonstrated ERα protein expression, while all 47 CGCG and all other giant cell containing bone tumours were negative. Most NOF samples had moderate staining intensity and between 24 and 49% of the spindle cells were ERα-positive. Our findings further support the role of endocrine regulation via oestrogen in the spontaneous regression in NOF. Whether oestrogen signalling at puberty is involved in the induction of senescence in the neoplastic cells of NOF harbouring RAS/MAPK pathway mutations needs further research. Since ERα expression was not observed in other giant cell containing bone lesions with overlapping morphological features, positive ERα expression may favour the diagnosis of NOF in challenging diagnostic cases.

## Introduction

Non-ossifying fibroma (NOF) and central giant cell granuloma (CGCG) are both benign lesions occurring in bone, presenting with highly similar histology. NOF is however predominantly located in the metaphyseal area of long bones and is more common in the first and second decade of life. In contrast, CGCG is located in the jaw bones and is more frequent in females aged under 30 years [1–5]. Most cases of NOF are asymptomatic and regress spontaneously after puberty. The majority of CGCG are slowly growing with mild clinical behaviour, while a minority of cases demonstrates a more aggressive clinical course characterised by rapid growth, cortical perforation, tooth displacement, root resorption and frequent recurrences after curettage. The general consensus is that CGCG always requires treatment either with local surgery, by using pharmacological treatment or a combination of both.

Recently, Baumhoer and colleagues identified KRAS, FGFR1 and NF1 mutations in 81% (48/59) of patients with NOF [4]. Interestingly, the morphology of NOF with spindle-shaped cells admixed with osteoclast-like giant cells is similar to the morphology observed in CGCG. Importantly, Gomez et al. showed that 72% of CGCG harbour similar RAS-MAPK pathway activating mutations including TRP4, KRAS and FGFR1, suggesting a pathogenetic relationship to NOF [3].

We hypothesised that the observed spontaneous regression in NOFs during puberty may be a result of increased endocrine regulation by oestrogen [1]. Therefore, we determined ERα expression by immunohistochemistry in NOF, CGCG and other giant cell containing bone lesions with overlapping morphological features in order to explain this distinct clinical feature in the background of a shared pathogenetics.

## Materials and methods

### Samples

We were able to retrieve formalin-fixed paraffin-embedded specimens from 16 biopsies of NOF cases (diagnosed between 2007 and 2019) from the archive in the Department of Pathology, Leiden University Medical Center (LUMC). The diagnosis was made in conjoint assessment by radiologists and pathologists.

In total, 47 formalin-fixed paraffin-embedded tissue samples from clinically, histologically and radiologically confirmed CGCG cases were used. In all these patients, a hyperparathyroidism was excluded by standard laboratory investigations and none was diagnosed with a CGCG-related syndrome (Noonan syndrome, neurofibromatosis, cherubism). Tissue samples from 6 of the 47 CGCG cases were retrieved from the archive of the LUMC pathology department as biopsy material on whole slide. The tissue samples from the 41 remaining CGCG cases were retrieved as an existing tissue micro array (TMA) from the department of Oral and Maxillofacial Surgery of the Amsterdam UMC. From 7 of these 41 CGCG cases, we also used whole slide to compare result between whole slide and TMA results. The TMA was constructed with 1-mm tissue cores from decalcified formalin-fixed paraffin-embedded tissue of 41 CGCGs. These tumours were originally diagnosed and treated between 1994 and 2014. All material was handled according to the ethical guidelines described in the Code for Proper Secondary Use of Human Tissue in The Netherlands. The TMA paraffin block was subsequently sliced into 4–6-μm-thick sections in order to make slides.

Furthermore, we included previously published TMAs from other giant cell containing bone tumours [6], including giant cell tumour of bone (*n* = 75), chondroblastoma (*n* = 12), chondromyxoid fibroma (*n* = 12), aneurysmal bone cyst (*n* = 6), and telangiectatic osteosarcoma (*n* = 6).

### Immunohistochemistry

Immunohistochemical staining was performed using ERα antibody (clone EP1, DAKO, Glostrup, Denmark). For immunohistochemical staining, slides were first deparaffinised and rehydrated using xylene and graded concentration of ethanol, respectively. Endogenous peroxidase was blocked in 0.3% H2O2 solution; subsequently, antigen retrieval was performed using Tris–EDTA pH 9.0 and microwaved. After a blocking step, using 30 min 5% non-fat dry milk in PBS/1%BSA, the slides were incubated overnight at 4 °C with ERα antibody, diluted 1:400 in PBS/BSA 1%. The ERα antibody was detected and visualised with BrightVision, 1 step detection anti-mouse/rabbit HRP (ImmunoLogic, Duiven, Netherlands) and DAB + substrate chromogen system (DAKO, Glostrup, Denmark). Lastly, haematoxylin was used to counterstain the slides, after which the slides were dehydrated and mounted using micromount (Leica Microsystems, Wetzler, Germany). For the negative control, the first antibody was excluded and as a positive control, a whole slide with a breast adenocarcinoma was included.

ERα expression in the samples was scored by evaluating staining intensity and the percentage of positively stained cells by two observers (AC, EG). The samples were assigned a score of 0, negative; 1, weak; 2, moderate or 3, strong, to indicate staining intensity, in which only nuclear staining was considered. The scores for the percentage of ERα positive cells were determined as 0 = 0%, 1 = 1–24%, 2 = 25–49%, 3 = 50–74% and 4 = 75–100% [6].

The samples were subdivided into ERα positive or ERα negative. ERα positive was defined as an overall staining score > 0 and negative as a score of 0, in which the overall staining score is the sum of the score for staining intensity and percentage of positively stained cells.

## Results

The median age of our NOF cohort was 16 years with 68% males and 32% females (see Table 1). NOF was located in the tibia, fibula or femur.

**Table 1:** Clinical characteristics of study cases

ID	Bone location	Age	Gender
*NOF_01*	Tibia	10	Female
*NOF_02*	Tibia	14	Male
*NOF_03*	Fibula	40	Male
*NOF_04*	Femur	29	Male
*NOF_05*	Femur	23	Male
*NOF_06*	Tibia	17	Male
*NOF_07*	Femur	10	Female
*NOF_08*	Tibia	19	Male
*NOF_09*	Femur	12	Male
*NOF_10*	Tibia	16	Female
*NOF_11*	Tibia	14	Male
*NOF_12*	Femur	13	Male
*NOF_13*	Tibia	16	Female
*NOF_14*	Tibia	5	Male
*NOF_15*	Tibia	21	Male
*NOF_16*	Fibula	18	Female
*CGCG_01*	Maxilla	12	Male
*CGCG_02*	Maxilla	19	Male
*CGCG_03*	Maxilla	17	Male
*CGCG_04*	Mandibula	9	Male
*CGCG_05*	Maxilla	21	Male
*CGCG_06*	Maxilla	4	Male
*CGCG_07*	Maxilla	13	Male
*CGCG_08*	Mandibula	56	Male
*CGCG_09*	Mandibula	6	Male
*CGCG_10*	Maxilla	14	Male
*CGCG_11*	Maxilla	12	Male
*CGCG_12*	Maxilla	12	Male
*CGCG_13*	Mandibula	6	Male
*CGCG_14*	Mandibula	46	Male
*CGCG_15*	Maxilla	3	Male
*CGCG_16*	Mandibula	12	Male
*CGCG_17*	Mandibula	7	Male
*CGCG_18*	Mandibula	13	Male
*CGCG_19*	Mandibula	32	Female
*CGCG_20*	Mandibula	29	Female
*CGCG_21*	Mandibula	52	Female
*CGCG_22*	Mandibula	42	Female
*CGCG_23*	Mandibula	30	Female
*CGCG_24*	Maxilla	13	Female
*CGCG_25*	Maxilla	24	Female
*CGCG_26*	Maxilla	24	Female
*CGCG_27*	Mandibula	15	Female
*CGCG_28*	Mandibula	46	Female
*CGCG_29*	Mandibula	47	Female
*CGCG_30*	Mandibula	37	Male
*CGCG_31*	Mandibula	12	Male
*CGCG_32*	Mandibula	13	Male
*CGCG_33*	Mandibula	36	Male
*CGCG_34*	Mandibula	49	Female
*CGCG_35*	Mandibula	15	Female
*CGCG_36*	Mandibula	16	Female
*CGCG_37*	Maxilla	45	Female
*CGCG_38*	Maxilla	13	Female
*CGCG_39*	Maxilla	15	Female
*CGCG_40*	Mandibula	50	Female
*CGCG_41*	Maxilla	21	Female
*CGCG_42*	Mandibula	40	Female
*CGCG_43*	Mandibula	41	Female
*CGCG_44*	Mandibula	54	Male
*CGCG_45*	Mandibula	61	Female
*CGCG_46*	Mandibula	7	Male
*CGCG_47*	Mandibula	40	Male

The median age of CGCG patients was 21 years (53% males and 47% females) and the tumours were located in the maxilla or mandible.

**Fig. 1:**
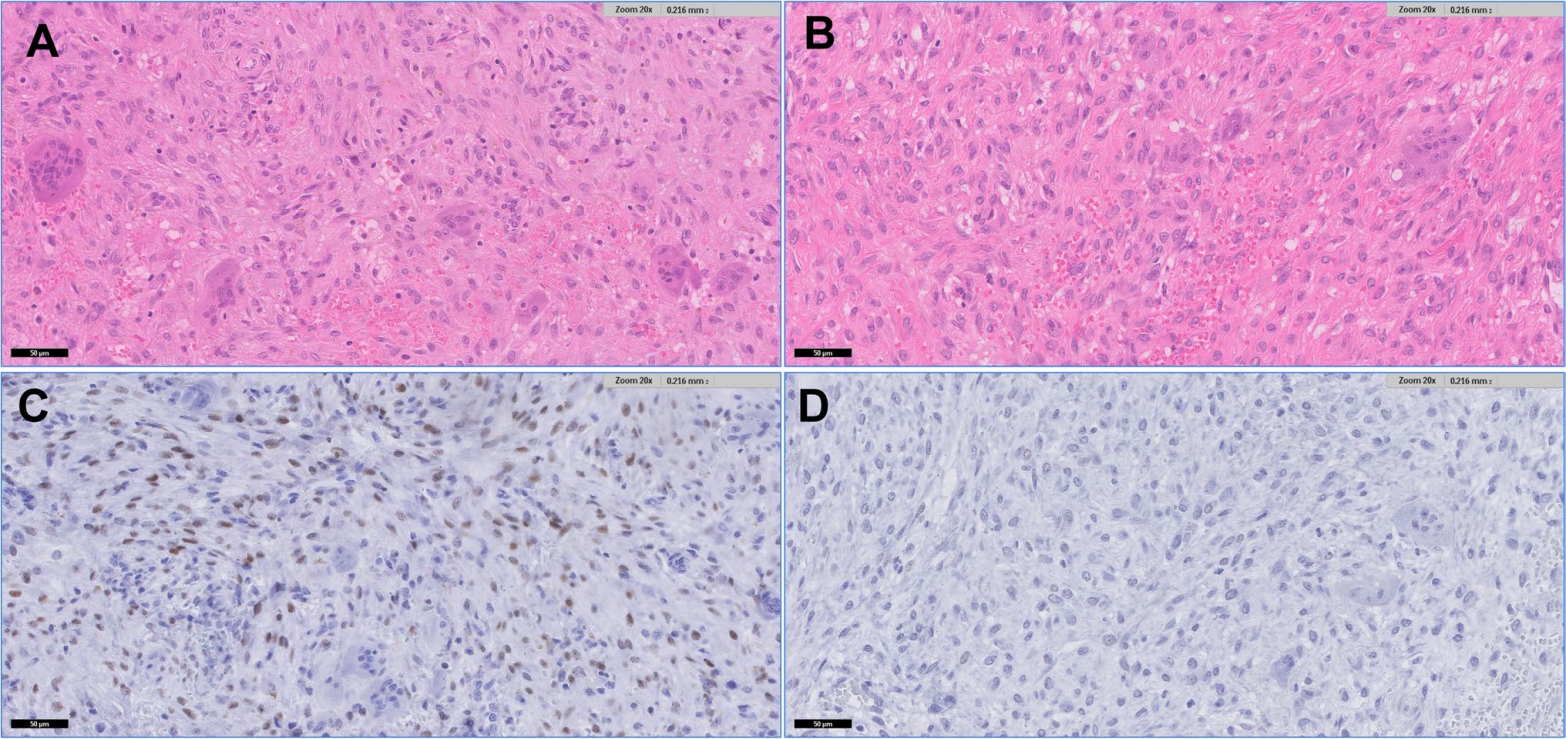
Morphology and ERα staining in non-ossifying fibroma (NOF) and central giant cell granuloma (CGCG). **A** H&E showing typical morphology of NOF with bland spindle shaped cells with oval and elongated nuclei with fine chromatin pattern, intermixed with osteoclast type giant cells. **B** NOF is indistinguishable microscopically from CGCG with similar morphological findings. **C** Positive nuclear ERα expression in a proportion of the spindle-shaped cells in NOF while **D** ERα expression is absent in CGCG

We found that all NOF cases showed moderate expression of ERalpha in 24–49% in the bland spindle–shaped cells with oval and elongated nuclei which were intermixed with ERalpha negative osteoclast-like giant cells (Fig. 1A-C). We did not observe differences in staining intensity or percentage of ER alpha expression between male and female patients with NOF. In contrast, all CGCG lesions were negative (Fig. 1B-D): all spindled cells and multinucleated giant cells did not stain for ERα expression in CGCG cases on TMA as well as whole-slide cases.

All other giant cell containing bone tumours, including giant cell tumour of bone, chondroblastoma, chondromyxoid fibroma, aneurysmal bone cyst and telangiectatic osteosarcoma, were also completely negative for ERα expression.

## Discussion

Our data support the hypothesis that increased endocrine regulation by oestrogen signalling through the ERalpha receptor during puberty is involved in the spontaneous regression of NOFs. Despite identical histology and molecular aberrations, spontaneous regression is not observed in CGCG and here, we show that ERalpha expression is lacking in CGCG.

Thus, similar oestrogen-regulated senescence occurring in the growth plate during puberty might also be initiated in the dispersed mutant cells in NOF lesions, as previously hypothesised [7]. This would result in a decline in mutant cells and lack of reciprocal signalling between mutant and WT cells disturbing the so-called landscaping effect. Reciprocal signalling is important for progression of the lesion and maintaining the microenvironment of the lesion, while disturbance of the balance between WT and mutant cells in NOF might eventually lead to regression.

Almost 69% of the NOF patients are younger than 18 years in our study; however, a significant proportion of patients with CGCG is also aged below 18 (51%). Even in patients aged below 18 with CGCG, no expression of ERα was observed, indicating that rather tumour-specific micro-environmental factors play a role instead of age. The absence of ERα expression in CGCG is in concordance with a study performed by Whitaker et al. in 10 cases of CGCG [8].

Since CGCG are exclusively located in the maxillofacial bone, it is tempting to speculate. The role of oestrogen in different stages of bone development is complex and may be different between maxillofacial bones derived from intramembranous versus long bones derived from endochondral ossification.

NOF not only shows overlapping morphological features with CGCG; the histology comprised of spindle shaped cells admixed with osteoclast-like giant cells may also overlap with other giant cell containing bone tumours such as giant cell tumour of bone, chondroblastoma, chondromyxoid fibroma, aneurysmal bone cyst and giant cell rich or teleangiectatic osteosarcoma. Although correlation of morphology with the radiological differential diagnosis remains the cornerstone in diagnostic decision-making, additional immunohistochemical or molecular diagnostic markers are helpful in challenging cases with overlapping morphological features especially in scarce biopsy material. Based on our results, the finding of positive ERα expression could favour the diagnosis for NOF above other giant cell containing bone lesions after thorough radiological correlation.

Nevertheless, caution should be taken using ERalpha expression as a diagnostic marker because of conflicting results when different antibody clones are used as was shown in several studies performed in breast cancer [9]. A study performed by Olivera et al. [10] showed positive staining for ER (clone 1D5) in 51% of 88 giant cell tumour of bone cases and a study performed by Romeo et al. [11] showed positive ERalpha expression (clone ESR1) in all tested chondroblastomas (*n* = 15). We used a different antibody (EP1) to determine ER expression, which may explain these conflicting results.

A limitation of our study is the fact that NOFs were mostly stained on whole slides, while the CGCG and other giant cell containing tumours were stained on tissue microarray. We still believe that the results are reliable since we included whole tissue slides of 7 cases of CGCG that were also included in the TMA which showed identical results; therefore, excluding false-negative IHC results due to scarce tumour material in these cases in our series of CGCG. Additionally, the TMAs including GCTB cases were positive for other antibodies [6].

In conclusion, our findings support the role of endocrine regulation via oestrogen in the spontaneous regression in NOF, which is not observed in CGCG or other giant cell containing bone lesions. The exact role of ERα in the spontaneous regression during puberty of NOF remains uncertain and further elucidation of the mechanism by which regression is induced might reveal new therapeutic options for other RASopathies. Since ERα expression was not observed in other giant cell containing bone lesions with overlapping morphological features, positive ERα expression (clone EP1) may favour the diagnosis of NOF in challenging diagnostic cases.

## References

[1] Bovee JV, Hogendoorn PC (2019). Non-ossifying fibroma: A RAS-MAPK driven benign bone neoplasm. J Pathol.

[2] Gomes CC, Gayden T, Bajic A, Harraz OF, Pratt J, Nikbakht H (2018). TRPV4 and KRAS and FGFR1 gain-of-function mutations drive giant cell lesions of the jaw. Nat Commun.

[3] Gomes CC, Gomez RS (2019). MAPK pathway-activating mutations drive giant cell lesions of the jaws and non-ossifying fibromas of bone. J Pathol.

[4] Baumhoer D, Kovac M, Sperveslage J, Ameline B, Strobl AC, Krause A (2019). Activating mutations in the MAP-kinase pathway define non-ossifying fibroma of bone. J Pathol.

[5] de Lange J, van den Akker HP, van den Berg H (2007). Central giant cell granuloma of the jaw: a review of the literature with emphasis on therapy options. Oral Surg Oral Med Oral Pathol Oral Radiol Endod.

[6] Cleven AH, Hocker S, Briaire-de Bruijn I, Szuhai K, Cleton-Jansen AM, Bovee JV (2015). Mutation analysis of H3F3A and H3F3B as a diagnostic tool for giant cell tumor of bone and chondroblastoma. Am J Surg Pathol.

[7] Boon E, van der Graaf WT, Gelderblom H, Tesselaar ME, van Es RJ, Oosting SF (2017). Impact of chemotherapy on the outcome of osteosarcoma of the head and neck in adults. Head Neck.

[8] Whitaker SB, Bouquot JE (1994). Estrogen and progesterone receptor status of central giant cell lesions of the jaws. Oral Surg Oral Med Oral Pathol.

[9] Cheang MC, Treaba DO, Speers CH, Olivotto IA, Bajdik CD, Chia SK (2006). Immunohistochemical detection using the new rabbit monoclonal antibody SP1 of estrogen receptor in breast cancer is superior to mouse monoclonal antibody 1D5 in predicting survival. J Clin Oncol.

[10] Olivera P, Perez E, Ortega A, Terual R, Gomes C, Moreno LF (2002). Estrogen receptor expression in giant cell tumors of the bone. Hum Pathol.

[11] Romeo S, Szuhai K, Nishimori I, Ijszenga M, Wijers-Koster P, Taminiau AH (2009). A balanced t(5;17) (p15;q22–23) in chondroblastoma: frequency of the re-arrangement and analysis of the candidate genes. BMC Cancer.

